# Expression patterns of *Passiflora edulis APETALA1*/*FRUITFULL* homologues shed light onto tendril and corona identities

**DOI:** 10.1186/s13227-017-0066-x

**Published:** 2017-02-02

**Authors:** Livia C. T. Scorza, Jose Hernandes-Lopes, Gladys F. A. Melo-de-Pinna, Marcelo C. Dornelas

**Affiliations:** 1 Departamento de Biologia Vegetal, Instituto de Biologia, Universidade Estadual de Campinas, Rua Monteiro Lobato, 255, 13083-862 Campinas, SP Brazil; 2 Institute of Molecular Plant Sciences, University of Edinburgh, Max Born Crescent, King’s Buildings, Edinburgh, EH9 3BF UK; 3 Departamento de Botânica, Instituto de Biociências, Universidade de São Paulo, Rua do Matão 277, 05508-090 São Paulo, SP Brazil

**Keywords:** *APETALA1*, *FRUITFULL*, Corona, MADS-box, Flower meristem, *Passiflora edulis*, Passifloraceae, Tendril

## Abstract

**Background:**

*Passiflora* (passionflowers) makes an excellent model for studying plant evolutionary development. They are mostly perennial climbers that display axillary tendrils, which are believed to be modifications of the inflorescence. Passionflowers are also recognized by their unique flower features, such as the extra whorls of floral organs composed of corona filaments and membranes enclosing the nectary. Although some work on *Passiflora* organ ontogeny has been done, the developmental identity of both *Passiflora* tendrils and the corona is still controversial. Here, we combined ultrastructural analysis and expression patterns of the flower meristem and floral organ identity genes of the MADS-box *AP1*/*FUL* clade to reveal a possible role for these genes in the generation of evolutionary novelties in *Passiflora*.

**Results:**

We followed the development of structures arising from the axillary meristem from juvenile to adult phase in *P. edulis*. We further assessed the expression pattern of *P. edulis AP1*/*FUL* homologues (*PeAP1* and *PeFUL*), by RT-qPCR and in situ hybridization in several tissues, correlating it with the developmental stages of *P. edulis*. *PeAP1* is expressed only in the reproductive stage, and it is highly expressed in tendrils and in flower meristems from the onset of their development. *PeAP1* is also expressed in sepals, petals and in corona filaments, suggesting a novel role for *PeAP1* in floral organ diversification. *PeFUL* presented a broad expression pattern in both vegetative and reproductive tissues, and it is also expressed in fruits.

**Conclusions:**

Our results provide new molecular insights into the morphological diversity in the genus *Passiflora*. Here, we bring new evidence that tendrils are part of the *Passiflora* inflorescence. This points to the convergence of similar developmental processes involving the recruitment of genes related to flower identity in the origin of tendrils in different plant families. The data obtained also support the hypothesis that the corona filaments are likely *sui generis* floral organs. Additionally, we provide an indication that *PeFUL* acts as a coordinator of passionfruit development.

**Electronic supplementary material:**

The online version of this article (doi:10.1186/s13227-017-0066-x) contains supplementary material, which is available to authorized users.

## Background

**Fig. 1:**
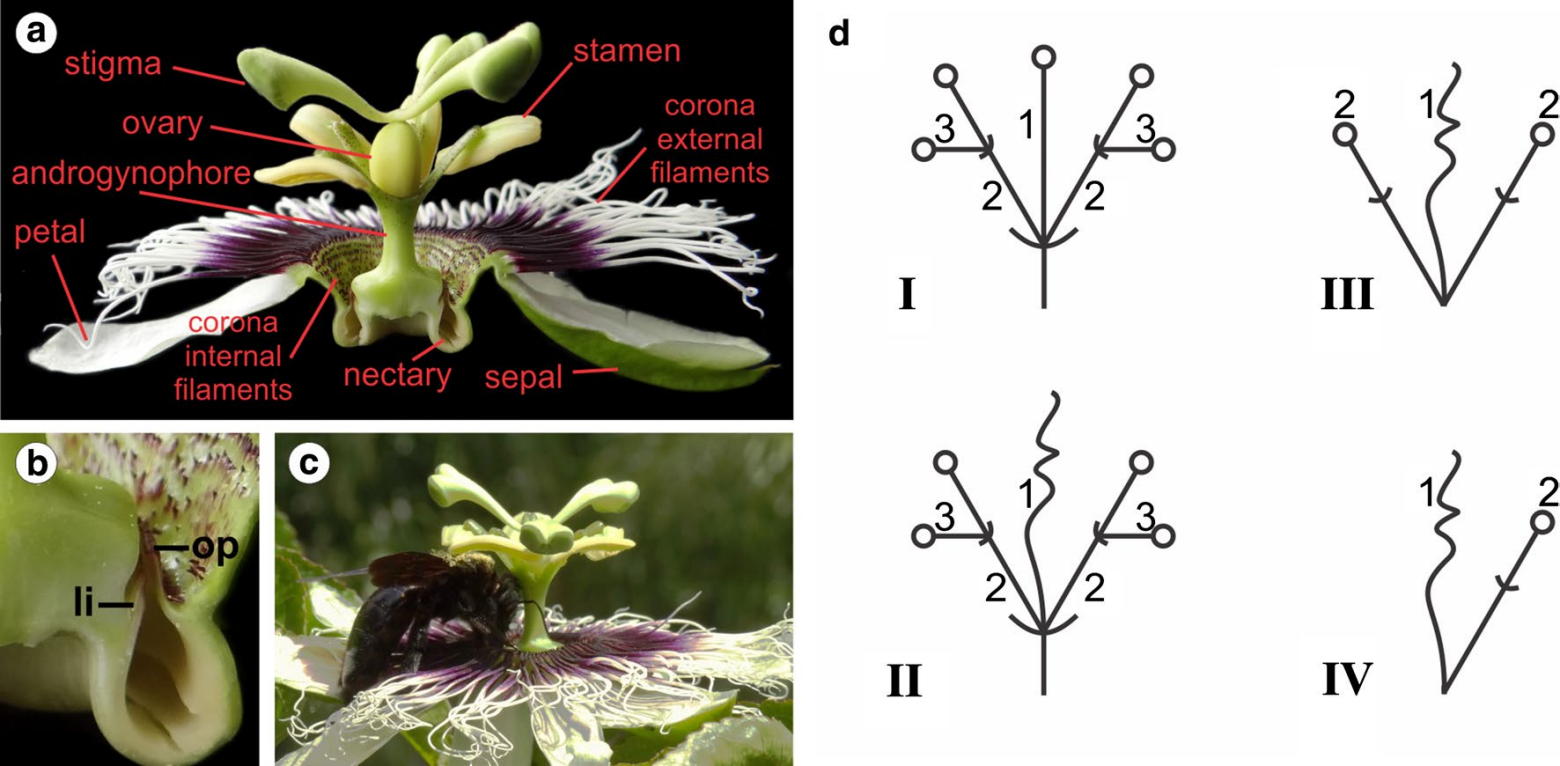
**a** Longitudinal section of a *P. edulis* flower, showing the floral organs and the nectary. **b** Magnification of the area of the nectary, where the operculum (*op*) and the limen (*li*) can be visualized enclosing the nectary chamber. **c** A carpenter bee (*Xylocopa* sp.), the *P. edulis* pollinator, landed on the corona to collect nectar. **d** Inflorescence models for Passifloraceae based on Krosnick and Freudenstein [9]. The general inflorescence type in the family is a compound cyme (*I*). The first-order axis (*1b*) may terminate in a flower (*I*) or a tendril (*II*). Different degrees of reduction of the inflorescence can be observed. In the genus *Passiflora,* most of the species have very reduced peduncles (*III* and *IV*), which is recognized by the retention of the tendril between the two flowers (*III*). In the case of *P. edulis*, there is only one second-order axis (*2*), which terminates in a single flower

One of the ways of understanding the origins of plant diversity is to look at the developmental processes regulating morphological innovations in different plant groups. The genus *Passiflora* (Passifloraceae) presents a good model for studying plant evolutionary biology, because of its unique features and the huge diversity of organ colours, sizes and shape found within the genus [1]. Passionflowers, the common given name for *Passiflora,* are in general vines that display axillary tendrils that coil around neighbouring branches for support and eventually reach areas with more light availability [1]. Tendrils are an example of convergent evolution and are present in several plant groups as derivatives of leaves (e.g., *Pisum sativum*, Fabaceae), lateral branches (e.g., *Echinocystis lobata*, Cucurbitaceae) or inflorescences (e.g., *Vitis vinifera*, Vitaceae) [2–5]. Interestingly, flowers and tendrils share the same ontogenetic programme in *Passiflora*, as each axillary meristem produces one tendril and one or more flowers flanking the tendril during the reproductive phase [6–8]. In *Passiflora edulis*, commercially grown for its edible fruits, the axillary meristems divide in two domes to form one tendril and one flower, the latter being subtended by three floral bracts [6, 7, 9]. One interpretation for the *Passiflora* tendril is that this structure is a modification of the first-order axis of a reduced compound cyme (Fig. 1d) [9]. According to this interpretation, together with the ontogenesis of tendrils and flowers [6, 7], it is reasonable to hypothesize that the *Passiflora* axillary meristem is actually an inflorescence meristem and thus the tendril would be a modified flower. Another interesting observation is that in the “Passion Dream” *P. edulis* genotype, a tendril can produce organ primordia such as leaves under specific temperature conditions (34/10 °C day/night regimen) [7]. This, on the other hand, suggests that tendrils might be modified shoots that are normally incapable of producing lateral organs. There is also some evidence of *Passiflora* tendrils producing flower structures, but these are limited to book graphical illustrations and old descriptions of these plants [10–12]. Currently, the interpretation of the *Passiflora* tendril remains unresolved. A way to help elucidate this question is to understand the molecular mechanisms involved in the switch to the reproductive stage in *Passiflora*, and the genetic mechanisms underpinning the formation of tendrils and flowers in this genus.

Flowering is a key developmental transition in the angiosperms life cycle. The transition to the reproductive phase is controlled by both external and internal factors such as light, hormones, temperature and nutrients, that culminate in the expression of transcription factors activating the transition from vegetative to reproductive phase [13, 14]. When plants are ready to flower, the shoot meristems (shoot apical meristem and/or axillary meristems) switch from the vegetative state, which produces leaves as lateral organs, to the reproductive state in which inflorescence meristems will produce flower meristems by expressing floral identity genes [15, 16]. The flower meristems produce floral organs consuming all meristematic cells, thus making the flower meristem determinate [17, 18]. The MADS-box genes of the *APETALA1*/*FRUITFULL* (*AP1*/*FUL*) lineage are, for example, essential coordinators of the reprogramming of the shoot apical meristem to the flower meristem [19, 20]. Despite some exceptions (for example, *AP1* also controls photoperiodic seasonal growth in hybrid aspen trees [21]), the function of *AP1/FUL* genes in the identity of inflorescence and flower meristem is generally conserved in angiosperms [19, 22–31].

The *AP1*/*FUL* gene lineage is marked by several duplication events that resulted in three lineages: *euAP1*, *euFUL* and *FUL*-*like*. In *Arabidopsis*, *AP1 and FUL* and act redundantly to control the architecture of the inflorescence and the identity of the flower meristems by affecting the expression domains of other regulators of the flowering gene network [19, 26, 32]. *FUL* is expressed in the inflorescence meristem, whereas *AP1* is expressed in the flower meristem, repressing *FUL* expression [19, 33]. *AP1* and *FUL* also present roles beyond flower meristem identity specification. For example, *AP1* is required for the proper development of sepals and petals in *Arabidopsis*. The ABCE model of flower development postulates that the combinatorial expression of functional classes of transcription factors is required for proper floral organ development [34, 35]. The A-class genes specify sepal identity and are represented by *AP1* and *AP2*. The A-class genes interact with the B-class genes, represented by *APETALA3* (*AP3*) and *PISTILLATA* (*PI*) to confer petal identity. Additionally, the C-class gene *AGAMOUS* (*AG*) interacts with the B-class genes to promote stamen identity. Finally, *AG* alone determines carpel identity. Later, the E-class genes, which includes the *SEPALLATA 1*-*4* (*SEP1*-*4*) genes, were added to the model and are required for the specification of all floral organs, acting in a combinatorial way with A, B and C functions [36]. The A-function, which determines sepal and petal identity in *Arabidopsis*, is currently debated for other species, as mutations in *AP1* homologues in other species such as tomato and *Antirrhinum* do not have an impact on petal identity [25]. However, functional characterization of *AP1* genes in other species is not extensive. Additionally, *AP1* homologues are also sometimes expressed in other floral organs like stamen and carpels, but the function of *AP1* in these organs is not well defined [24, 37–39]. The eu*FUL* genes also have different roles in plant development, being generally involved in the development of leaves, carpels and fruits, and also the maturation process of fleshy fruits [40–43].

Flower morphology varies greatly in angiosperms, and many plants have evolved more than four whorls of floral organs, presenting complex and specialized flowers. The emergence of such flower features is tightly related to the evolutionary adaptation to their pollination strategy. This is also the case for *Passiflora* flowers. Besides the sepals and petals, *Passiflora* flowers have one or more extra whorls of corona filaments located between the perianth and the androgynophore—a fusion of the androecium and gynoecium (Fig. 1a) [1]. The presence of the corona has great significance for the reproductive success of passionflowers as these organs, which vary greatly in number, size and colour among species, can function as a nectar guide, as a landing platform for insects (Fig. 1c), and even as a floral tube adapted to hummingbird pollination in species like *Passiflora mixta* [1, 44].

In *P. edulis* the corona is formed by a whorl of external filaments and several whorls of internal short filaments (Fig. 1a) [1, 45]. The inner border of the corona ends with two membranes named operculum and limen that together enclose a nectary chamber (Fig. 1b) [1]. The identity of the corona is not clear and the literature currently presents three hypotheses: the corona filaments might be modified stamens, modified petals or they might be truly novel floral structures [8, 45–47]. The expression of B- and C-class genes were analysed during *Passiflora caerulea* floral organ development, but *AP1* was not taken into account in the analysis [47]. Although some work has been done in order clarify the identity of these floral structures, the molecular mechanisms behind such structural innovations are still poorly explored.

Given the conserved nature of the MADS-box genes *AP1/FUL* in stabilising flower meristem identity [48, 49], we isolated one eu*AP1* and one eu*FUL* homologue in *P. edulis* and assayed their expression pattern by RT-qPCR and in situ hybridization. Our aim was to investigate the sites of expression of the putative homologues in *P. edulis* meristems and different plant tissues, such as the flower meristem, tendrils and floral organs, in order to help to shed light on tendril genetic identity. We evaluated the expression levels of these genes in different *P. edulis* tissues and organs, including shoot apices in either juvenile or reproductive phases, and followed the developmental stages of the axillary meristem and subsequent formation of tendrils and flowers by scanning electron microscopy. We used the site of expression of *PeAP1* and *PeFUL*, assessed by in situ hybridization, to suggest possible functions in identifying the axillary meristem as inflorescence or flower meristems and the tendril as a vegetative or reproductive organ or branch. Because *AP1*/*FUL* genes are also implicated in floral organ and fruit development in other species, we also explored the expression of *P. edulis* homologues during flower organ development to add new molecular knowledge to the formation of unusual flower organs, such as the corona filaments.

## Methods

### Plant material

Plants of *Passiflora edulis* Sims. f. *flavicarpa* Deg. were grown on soil in experimental fields or in greenhouses at the Plant Biology Department at the University of Campinas (UNICAMP), Campinas, São Paulo, Brazil. Alternatively, for root harvesting, plants were grown in hydroponics in the greenhouse.

### Scanning electron microscopy

*Passiflora edulis* shoot apices in the juvenile and adult phases were vacuum-infiltrated in 4% (v/v) paraformaldehyde in phosphate-buffered saline (PBS, 0.13 M NaCl, 7 mM Na_2_HPO_4_, 3 mM NaH_2_PO_4_, pH 7.0) and fixed at 4 °C overnight. The samples dehydrated in a series of increasing ethanol concentrations (25, 50, 75 and 95%), for 1 h each, and twice in 100% ethanol for 30 min. Samples were dried in a critical point dryer (CPD 030, Bal-Tec, Schalksmühle, Germany) and mounted on metal stubs. The dried mounted samples were partially dissected to show the structures hidden by leaf primordia, such as the axillary meristems, tendril primordia and flower meristems. The material was then sputter-coated with gold and examined in a LEO 435 VP electron microscope equipped with LEOUIF system for digital image acquisition.

### Identification of AP1 and FUL homologues in *P. edulis*

The search for *AP1* and *FUL* orthologs was performed using a RNA-seq database of leaves, flowers and colour break fruits of *Passiflora edulis cv. “Passion Dream,”* a hybrid of *P. edulis* Sims *f. flavicarpa* Deg and *P. edulis* Sims *f. edulis*. This database was produced and kindly made accessible by Dr. Alon Samach at The Robert H. Smith Institute of Plant Sciences and Genetics in Agriculture, in The Hebrew University of Jerusalem, Israel. The search was made using the standalone BLAST (blast 2.2.29) tool, and *AP1* and *FUL* protein sequences of *Arabidopsis thaliana* (AT1G69120 and AT5G60910, respectively) were used as queries. In order to ensure similarity, the *P. edulis* putative *AP1* and *FUL* homologue nucleotide sequences obtained were compared back to *Arabidopsis* proteins using BLASTX in TAIR10 protein. As the first hits obtained for the BLASTX were AP1 and FUL, respectively, we used these *P. edulis* sequences, which were named *PeAP1* (GenBank KY471457) and *PeFUL* (GenBank KY471458), for phylogenetic analysis. The identified *PeAP1* and *PeFUL* sequences can be found in the additional files [Additional file 1]. The putative *P. edulis* AP1 and FUL protein homologues were aligned with the “Clustal W” tool in the “MEGA6” software [51] together with other representative *AP1*, *FUL* and *FUL*-like protein homologues in other species. The *Arabidopsis* MADS-box proteins from the *SEPALLATA* (*SEP*) family were used as outgroup. The alignment was performed using the MADS, K, I, and C-terminal domains [29, 30, Additional file 2]. For the phylogenetic analysis, the best model for nucleotide substitution (JTT + I + G) was selected using the software “ProtTest 2.4” based on the lowest AIC score [50]. Phylogenetic analyses were run using maximum likelihood in “MEGA6” and the bootstrap support was calculated from 1000 replicates [51].

### Gene expression analysis by RT-qPCR

Total RNAs were isolated from different *P. edulis* tissues, including roots, stems (internodes), leaves, shoot apices in the juvenile phase (i.e., apices of one-month-old seedlings); shoot apices in the reproductive phase (i.e., 1-year-old plants producing tendrils and flower meristems), flower buds of different sizes (1–3 mm, 2 cm, 3–4 cm), bracts, flower organs dissected from flowers in anthesis including sepals, petals, corona filaments (including inner and outer filaments and the operculum), stamens, carpels and the androgynophore column. Additionally, total RNAs were also isolated from whole green fruits of approximately 4–5 cm in diameter and from pericarps of colour break (ripening) fruits. The mature leaves, tendrils and bigger flower buds were removed from the shoot apex samples. The RNA was isolated using TRIzol (Invitrogen, San Diego, CA) according to the manufacturer’s recommendations. For root and fruit material, the TRIzol was ineffective and RNA was isolated using the CTAB method as described by Chang et al. [52]. The RNA samples were treated with DNase using TURBO DNase (Ambion by Life Technologies, Carlsbad, CA) following manufacturer’s recommendations. After the RNA integrity was checked on a 1% agarose gel stained with ethidium bromide, 1 µg of RNA was used as a template for cDNA synthesis (SuperScript III™ First Strand Synthesis, Invitrogen, San Diego, CA). Primers for qRT-PCR were designed to amplify fragments in the C-terminus region of *PeAP1* and *PeFUL* genes, using Primer3Plus [53, Additional file 3: Table 1]. The primers were certified for amplification efficiency (*E*) using a cDNA dilution series as templates. The efficiency was calculated using the slope of the linear regression line generated in Microsoft Excel 2010 with the following equation: *E* = 10^[(−1/slope)−1]^ × 100 [Additional File 3: Table 1]. The specificity of each primer pair was verified by dissociation curve analysis (60–95 °C). Gene expression analysis was carried out using Platinum SYBR Green qPCR SuperMix-UDG (Invitrogen, San Diego, CA) in a Real-Time PCR System 7500 (Applied Biosystems, Foster City, CA). Relative expression levels were calculated based on the $2^{ - \Delta \Delta Ct}$ method [54]. P*eCAC* (GenBank KY471459) was used as the reference gene for expression normalization. The results presented are the mean ± standard deviation of three independent biological replicates.

### In situ hybridization

Gene-specific probe construction: DNA templates used for the synthesis of RNA probes were obtained by PCR amplification using a cDNA library from shoot apices in the reproductive phase. The amplified sequences had 559 and 615 bp for *PeAP1* and *PeFUL*, respectively [Additional file 3: Table 1]. To ensure specificity, the probe templates included the C-terminal domains for both genes. The antisense and sense probes were prepared using a DIG RNA Labelling Kit (SP6/T7; Roche) following the manufacturer’s recommendations. After synthesis, the probes were precipitated at −20 °C overnight using 3.8 M NH_4_Ac and ice-cold 100% ethanol (1:1:6 v/v), spun down, washed in ice-cold 70% ethanol, air-dried and resuspended in 50 µl of 0.1% DEPC-treated Milli-Q water. The probes were hydrolysed for 60 min at 60 °C with 1× volume of carbonate buffer (120 mM Na_2_CO_3_; 80 mM NaHCO_3_) and precipitated with 10 μl of 10% acetic acid, 12 μl of 3 M sodium acetate pH 4.8 and 312 μl of absolute ethanol at −20 °C overnight. Finally, the probes were spun down, washed in ice-cold 70% ethanol, air-dried and resuspended in 50 µl of DEPC-treated Milli-Q water. Probes were then used for hybridization.

*Hybridization*: Developing shoot apices in the reproductive phase, containing the apical meristem, the 5–6 leaf primordia and their respective axillary meristems, as well as flower buds in different developmental stages were collected from *P. edulis* plants. The biological material was fixed under vacuum in a freshly prepared solution of 4% paraformaldehyde (w/v) in PBS on ice under vacuum. After 15 min, the fixative solution was then renewed, and the samples were fixed overnight at 4 °C. Samples were subsequently washed in 0.85% NaCl (w/v) for 30 min and dehydrated in crescent ethanol series at 4 °C. The dehydrated material was transferred to ethanol/xylene solutions (3:1, 1:1, and 3:1, respectively) and then to pure xylene. Finally, the samples were embedded in Paraplast Plus (Sigma-Aldrich, St. Louis, MO) at 60 °C before being sectioned into 6–7 µm sections and mounted onto silanized slides. The pre-hybridization treatment consisted of: Paraplast Plus removal with two washes in pure xylene for 10 min each; re-hydration in a diluting ethanol series; one wash in PBS for 3 min; treatment with proteinase K (1 µg/ml) in Tris–EDTA (0.1 M Tris; 0.05 M EDTA) for 5 min at 37 °C; one wash in 0.2% glycine for 3 min; one wash in PBS for 3 min; fixation in 4% paraformaldehyde in PBS for 10 min; two washes in PBS; one wash in 0.5% acetic anhydride in 0.1% triethanolamine pH 8.0 for 10 min under constant agitation; one wash in PBS for 3 min; one wash in 0.85% NaCl for 1 min. The material was then dehydrated again in an ethanol series and let to air-dry. The hybridization buffer contained (for each slide): 15 µl of 10× salts (3 M NaCl; 0.1 M Tris–HCl pH 6.8; 0.1 M NaPO_4_; 50 mM EDTA); 60 µl of deionized formamide; 3 µl of Denhardt’s solution (1% Ficoll type 400, 1% polyvinylpyrrolidone and 1% BSA); 30 µl of 50% dextran sulphate; 12 µl of 0.1% DEPC-treated Milli-Q water; 2–4 µl of probe. The slides were placed in a humidified box with paper towels wet with autoclaved Milli-Q water. The hybridization buffer was poured onto the slides which were covered with Hybri-slips (Sigma-Aldrich) and kept at 50 °C overnight. The post-hybridization washes started with a brief wash in 2× SSC (0.3 M NaCl, 30 mM Na_3_C_6_H_5_O_7_) at room temperature to gently remove the coverslips. The slides were subsequently rinsed 3 times in 0.2× SSC at 55 °C for 25 min each rinse and then with a mix of 150 mM NaCl and 100 mM Tris–HCl pH 7.5 (buffer 1) for 5 min at room temperature. The slides were then incubated with gentle agitation for 1 h in 0.5% blocking agent (Roche, Basel, Switzerland) in buffer 1 followed by 30 min in 1% bovine serum albumin, 0.3% Triton X-100 in buffer 1 (this mix was named buffer 2). This was followed by a 1 h incubation in dilute antibody-conjugate anti-digoxigenin-AP (1:1000, Roche, Basel, Switzerland,) in buffer 2 and four washes of 25 min each in 0.3% Triton X-100 in buffer 1. Slides were briefly washed in buffer 1 and in 100 mM Tris–HCl pH 9.5, 100 M NaCl, 0.5 MgCl2 for 5 min each. The slides were kept in humidified box containing paper towels wet with autoclaved Milli-Q water, and 300 µl of NBT/BCIP solution (Amresco Inc. OH) were applied onto the slides. The slides were covered with coverslips and incubated overnight, protected from light. The sections where then observed and documented in a Zeiss Axiovert 35 microscope. Entellan (Merck, Billerica, MA, USA) was used as mounting medium for making permanent slides.

## Results

### Structural characterization of organ primordia development from juvenile to adult reproductive phases in *P. edulis*

**Fig. 2:**
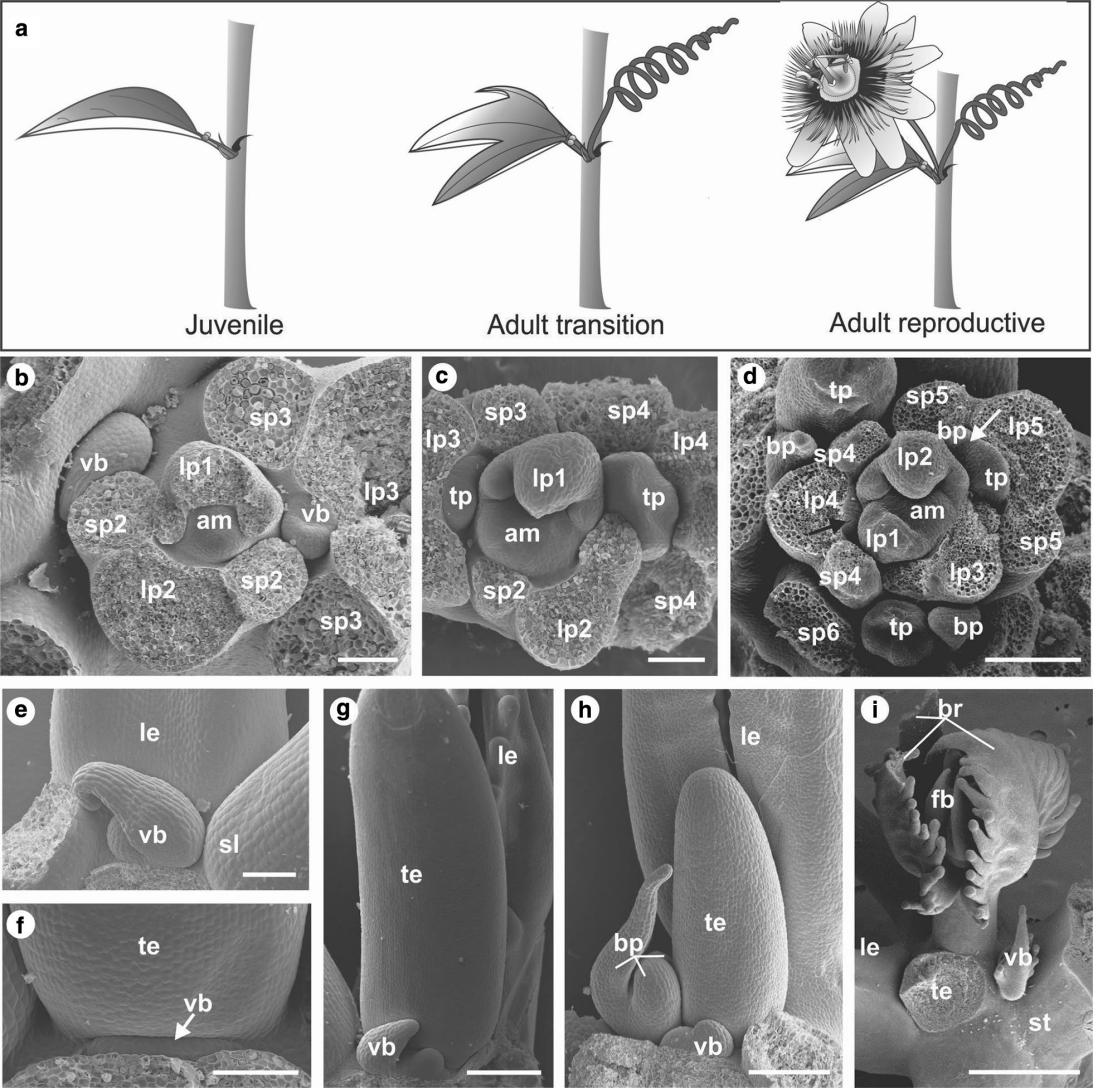
*Passiflora edulis* shoot apical behaviour. **a** Schematic representation of typical *P. edulis* phytomers in the juvenile, transition and reproductive stages. **b–i** SEM of *P. edulis* developing organs at different stages. **b** *Top view* of the shoot apex of a juvenile plant. Leaves primordia (l1–l3) develop in spiral phyllotaxy from the apical meristem (*am*) and are numbered according to the order they arise, so l1 is the youngest leaf. Two stipules (s1–s3) are formed laterally to each leaf primordium. A vegetative bud (*vb*) is observed in the axil of the third leaf (l3). **c** *Top view* of the apex of a plant in the adult transition stage, where the tendril primordium (*tp*) develops from the third leaf axillary meristem. **d** *Top view* of the apex of a plant in the adult reproductive stage. Here, the axillary meristem (*black arrow*), which appears as a *dome-shaped bump* in the axil of the fourth leaf (l4), will produce a tendril and a flower. In the fifth leaf (l5), we can observe the first bract primordium (*bp*) that covers the flower meristem (*white arrow*) and the tendril primordium (*tp*). **e** Details of the leaf (*le*) axil in the juvenile stage where only the vegetative bud develops (*vb*) in between the two stipules (*sl*). **f, g** Details of the vegetative bud developing in the region between the tendril (*te*) insertion and the stem in the adult transition stage. **h** Details of the leaf axil where the tendril (*te*), the flower (represented by the presence of three bracts *br*) and the vegetative bud (*vb*) developing in the reproductive stage. **i** The final position of the structures in the leaf (*le*) axil is visualized. The tendril (*te*) was removed to visualize the flower bud (*fb*) that is developing enclosed by the three bracts (*br*), and the vegetative bud (*vb*) is positioned between the flower-tendril complex and the stem (*st*) *Bars*: **b**, **c**, **f** = 100 µm; **d**, **e**, **g**, **h** = 200 µm; **i** = 1 mm

In *P. edulis*, the progression from the juvenile to the adult reproductive phase is macroscopically characterized by changes in leaf shape and the appearance of the tendril and the flower (Fig. 2a). Although *P. edulis* organ development in the reproductive stage was described previously [6, 7], information about organ primordia development encompassing the juvenile and transition phases remains undefined. In order to gain insights into structural appearance in the apices at each developmental stage, we characterized the ultrastructure of organ primordia development of *P. edulis* in detail at all stages.

In the juvenile phase, *P. edulis* plants produce lanceolate leaves with associated axillary meristems that further develop into vegetative buds and reiterate the plant growth pattern when activated (Fig. 2b, e). After the plants have produced 10–12 leaves, the axillary meristem produces one tendril in addition to the vegetative bud, morphologically marking the transition phase (Fig. 2c). During the transition phase, after the tendril primordium has developed, the vegetative bud grows from a group of cells between the adaxial side of the tendril and the stem (Fig. 2f, g). In the adult transition phase, only the tendrils and the vegetative bud develop from the axillary meristem, and the flowers are still absent (Fig. 2a, c, f, g). The number of nodes in the transition phase was 1–3, and this number varied among different plants. Although this phase is transitory, this indicates that the initial development of tendrils is not necessarily linked with flower development. However, when plants initiate the adult reproductive growth, the flower and the tendril originate from the same initial group of meristematic cells in the axillary meristem (Fig. 2d). While part of the axillary meristem will continuously grow and form a tendril, the other part will develop into one flower (Fig. 2d, h, i). The emergence of the flower is accompanied by the production of the first bract which covers the flower meristem and is followed by the development of two additional floral bracts (Fig. 2d, h, i). The vegetative bud develops only after the tendril and the first bract primordia have emerged, between the flower-tendril complex and the stem (Fig. 2h, i). After the plants have entered the adult reproductive phase, the axillary meristem will always produce one tendril and one flower.

The ultrastructural characterization of the organ primordia indicates that, although still partially autonomous events, the development of flowers is connected to the development of tendrils in *P. edulis.*

### Characterization of AP1/FUL genes from *P. edulis*

**Fig. 3:**
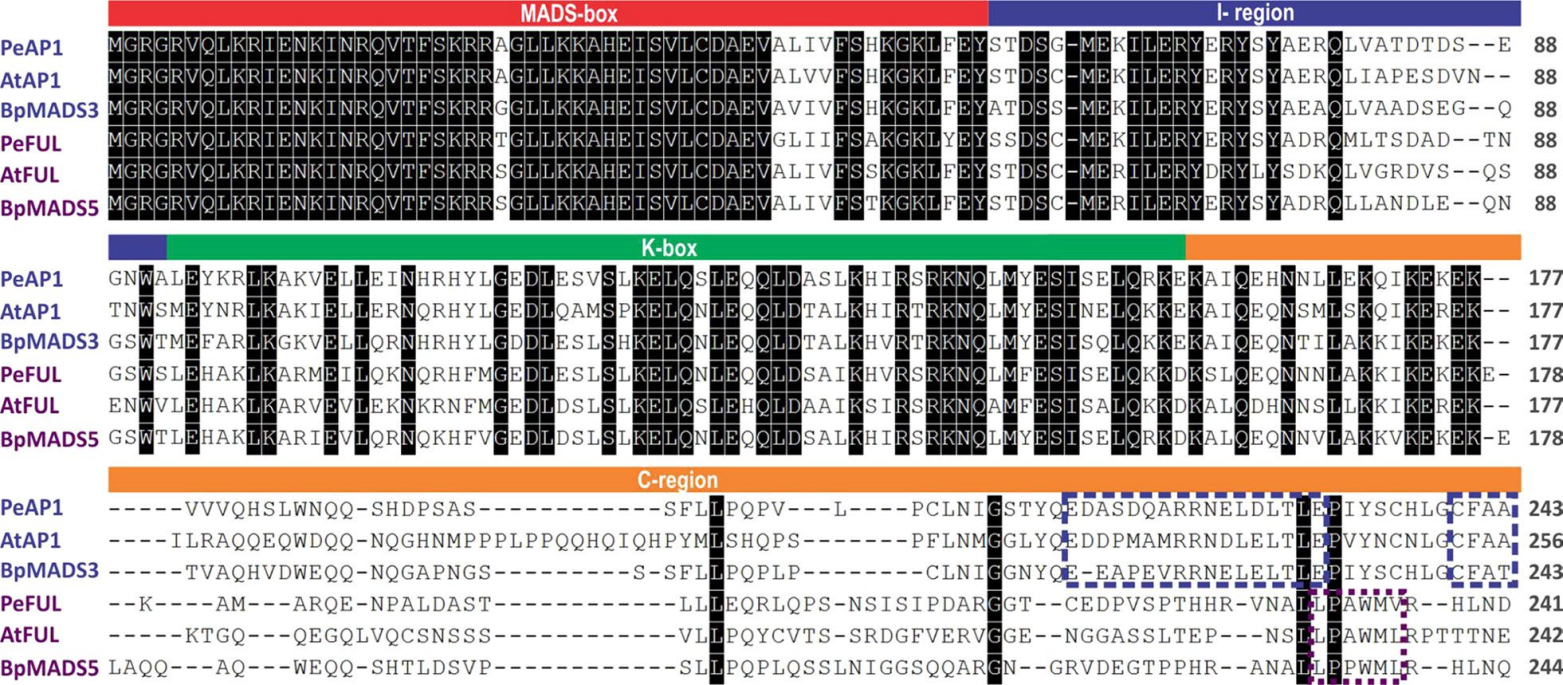
Comparative analysis of aligned eu*AP1* and eu*FUL* protein sequences of *A. thaliana* (AtAP1 and AtFUL), *P. edulis* (PeAP1 and PeFUL) and *B. pendula* (BpMADS3 and BpMADS5). The MADS-box protein motifs (MADS-box, I-region, K-box and C-region) are shown in the *boxes* above the aligned sequences. Conserved sites are shaded in *black*. The *blue dashed rectangles* at the C-region delineate the two eu*AP1* typical motifs (transcription activation domain and farnesylation motifs) and the *purple dashed rectangle* circumscribes the FUL-like motif [25]

Because the meristematic identity of the flower is determined by the MADS-box AP1/FUL genes, we selected *AP1* and *FUL* to investigate the identity of tendril and flower primordia in *P. edulis*. In order to identify putative *AP1* and *FUL* homologues for further genetic characterization, we blasted *A. thaliana AP1* and *FUL* sequences against a *P. edulis* RNA-seq database. The BLAST search resulted in a significant hit for each *AP1* and *FUL* gene. Both sequences contained an open reading frame with flanking 5′ and 3′ UTR regions with a total size of 1152 and 1171 bp for the putative *AP1* and *FUL* homologues, respectively [Additional file 1]. The search against the TAIR database by using the BLASTX tool and these *P. edulis* sequences as queries confirmed AP1 and FUL had the highest similarity score. The full-length coding sequence of the putative *P. edulis AP1* orthologue shares 75% of nucleotide identity and 70% amino acid identity with *Arabidopsis AP1*, while the putative *FUL* orthologue coding sequence shares 69% nucleotide identity and 64% amino acid identity with *A. thaliana FUL.* The sequences were then named *PeAP1* and *PeFUL*, according to their similarity to *A. thaliana AP1* and *FUL*, respectively. The predicted PeAP1 protein sequence has 243 amino acids, while the predicted PeFUL protein has 241 amino acids (Fig. 2). An analysis of these *P. edulis* predicted protein sequences aligned homologues in *A. thaliana* and *Betula pendula* (BpMADS3 and BpMADS5) identified a conserved region in the N-terminal part, which corresponds to the MADS-domain (Fig. 3). In the C-terminal end, two *euAP1* typical domains (transcription activation and farnesylation) were observed in PeAP1. Additionally, a typical domain present in *euFUL* sequences, comprised by six hydrophobic amino acids, was also present in PeFUL and absent in PeAP1 (Fig. 3) [25, 55].

**Fig. 4:**
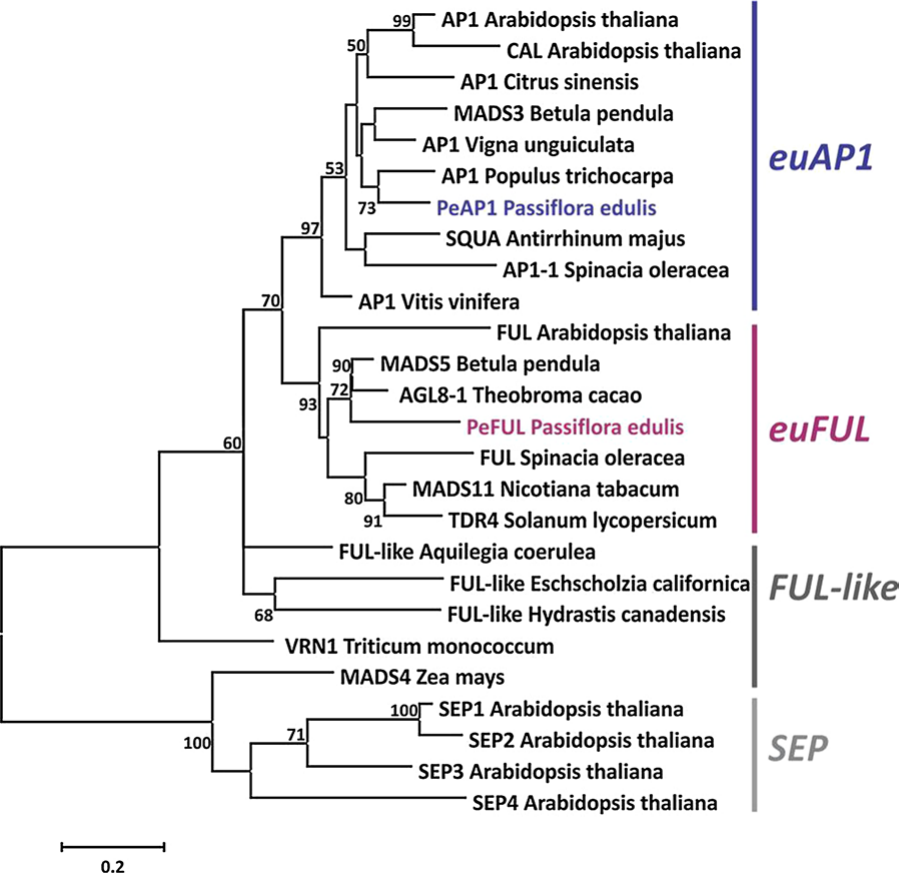
Maximum likelihood tree of protein sequences including PeAP1 and PeFUL. The tree presents PeAP1 and PeFUL featured in *blue* and *purple*, respectively, and other representative homologues of *AP1*, *FUL* and *FUL*-like of other species. SEP protein sequences are placed as outgroup. The accession numbers for the sequences are presented in the Additional file 2. The phylogenetic analysis was performed using the protein MADS-box, I-region, K-box and C-region. The eu*AP1*, eu*FUL*, *FUL*-like and *SEP* gene lineages are shown on the *right side* of the tree. Bootstrap values are shown at nodes

To clarify the relationship among PeAP1, PeFUL and other members of eu*AP1* and eu*FUL* clades, a phylogenetic analysis was performed using amino acid sequences of *AP1/FUL* clade from several plant species and *A. thaliana SEP*s as an outgroup (Fig. 4). The maximum likelihood tree generated three clades that corresponded to *euAP1*, eu*FUL*, and *SEP* and a paraphyletic group formed by *FUL*-like genes (Fig. 4). PeAP1 was clustered together with a *Populus trichocarpa* sequence in the eu*AP1* clade, which also included *A. thaliana* AP1 and *A. majus* SQUA proteins. PeFUL grouped with *Theobroma cacao* (cacao) and *B. pendula* (silver birch) FUL genes, in the eu*FUL* clade where *A. thaliana* FUL was also present, confirming that of PeAP1 and PeFUL are eu*AP1* and eu*FUL* sequences, respectively (Fig. 4).

Overall, the data establishes PeAP1 and PeFUL as homologues of AtAP1 and AtFUL, respectively, with critical similarities in gene and protein regulatory domains. Therefore, we analysed the expression patterns of *PeAP1* and *PeFUL* to see if there was a correlation with the expression pattern and the development of meristems and floral organs.

### *PeAP1* is expressed in both the flower meristem and tendril primordium, as well as in the flower perianth, including the corona

**Fig. 5:**
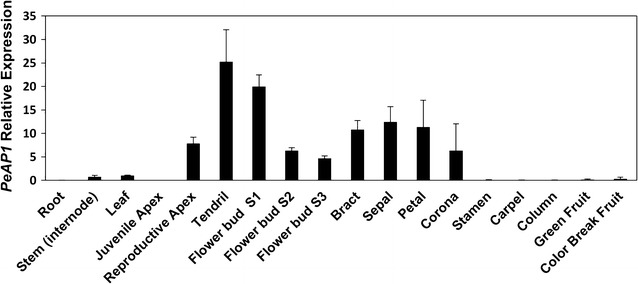
Expression profiles of *PeAP1* in different *P. edulis* vegetative and reproductive tissues by RT-qPCR. The expression was normalized using the expression of the constitutive *PeCAC* gene. The *bars* refer to the standard error of three biological replicates

First, we assessed the expression of *PeAP1* by RT-qPCR. Expression of the *PeAP1* gene was highly activated in apices in the adult reproductive phase, compared to apices in the juvenile phase (Fig. 5). As anticipated, flower buds, especially in their initial phase of development, showed high expression of *PeAP1*, as *AP1* is required during initial steps of flower development in other species. Notably, we found that *PeAP1* expression is not only activated in bracts, sepals and petals, but also in corona filaments, suggesting that the corona share a similar genetic programming with perianth organs. Surprisingly, however, tendril displayed the highest *PeAP1* expression among all the structures analysed. By contrast, the vegetative tissues (root, stem and leaves) as well as mature stamens, carpels, androgynophore column and both green and maturing fruits did not show considerable expression of *PeAP1* in the RT-qPCR analysis (Fig. 5).

**Fig. 6:**
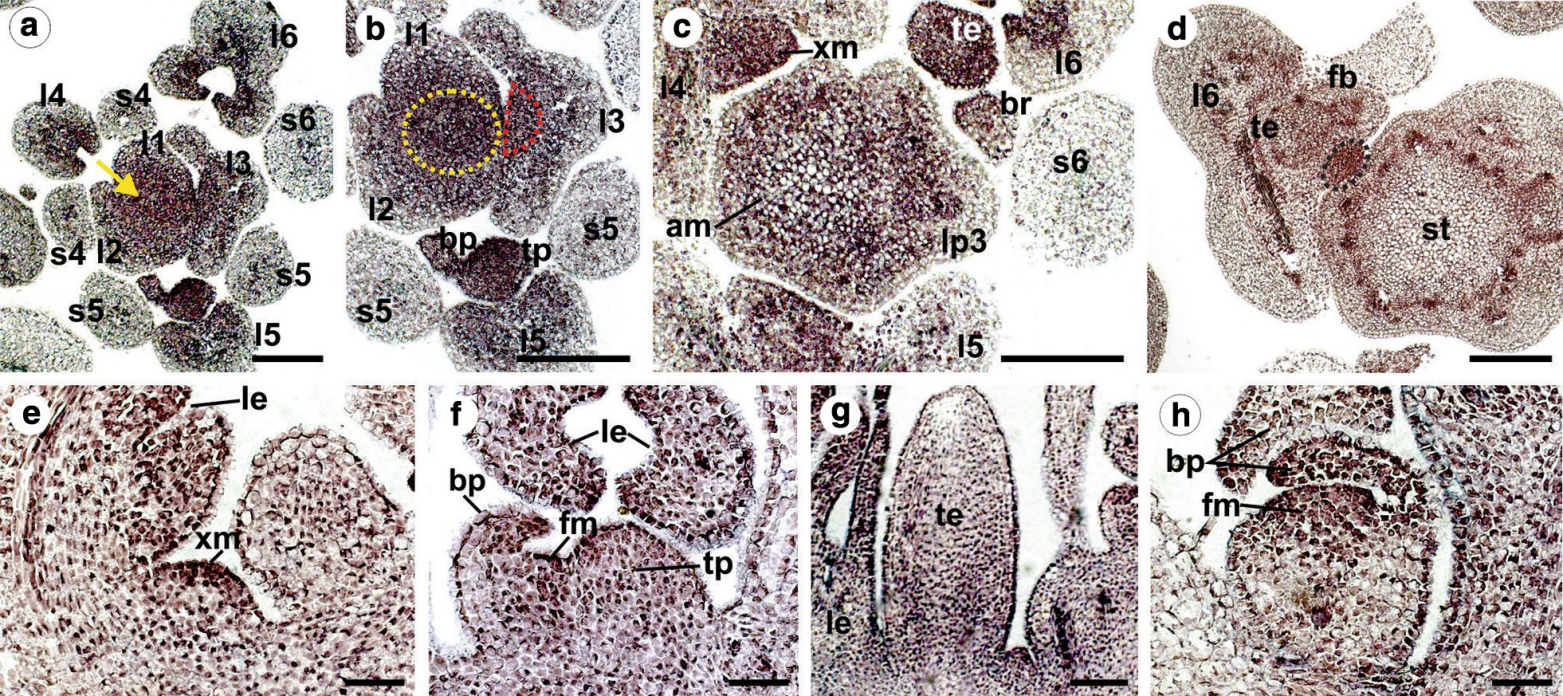
*PeAP1* expression pattern by in situ hybridization of shoot apices of plants in the reproductive stage. **a–d** Cross sections of the *P. edulis* shoot apex from top to bottom. *PeAP1* expression is detected in the adaxial side of the young leaves (l1–6), in the apical meristem (*yellow arrow* in **a** and *yellow dashed circle* in **b**), in the axillary meristem (*dashed red semicircle* in the third leaf primordium in **b** and “xm” in **c**), and in the tendril and bract primordia (*tp* and *bp,* respectively). *PeAP1* transcripts were not detected in stipules (*s*). In **d**, *PeAP1* is detected in the developing vegetative bud (*black dashed circle*), located between the tendril–flower complex (*te* and *fb*) and the stem (*st*). As sections distance from the apical meristem to the stem, *PeAP1* expression in this region becomes fainter and it is not evident in the differentiated stem (*st* in **d**). **e** *PeAP1* expression in axillary meristem (*xm*) in longitudinal section. *le* Leaf. **f** Longitudinal section of a tendril primordium and emerging flower meristem. *PeAP1* transcripts are detected in the adaxial region of the bract primordium (*bp*), in the floral meristem (*fm*) and in the tendril primordium (*tp*). **g** Longitudinal section with *PeAP1* transcripts detected in both young leaf (*le*) and tendril (*te*). **h** Expression of *PeAP1* in a young flower bud, showing transcripts in the floral meristem (*fm*) and in bract primordia (*bp*). *Bars*: **a**, **b**, **c**, **d** = 100 µm; **e**, **f**, **g**, **h** = 50 µm

To gain further insights into the timing and tissue-specific localization of *PeAP1* expression in the initial stages of tendril and flower development, we performed in situ hybridization targeting *PeAP1*. In the reproductive apices, we found that *PeAP1* was expressed in both apical and axillary meristems (Fig. 6a–c, e), with reducing activation in apical meristems in the stem, as indicated by reduction in the hybridization signal (Fig. 6d). *PeAP1* transcripts were observed in the axillary meristems from their initial formation until they divided to form the tendril and the flower (Fig. 6b–f). In the initial stages of flower development, *PeAP1* transcripts were detected in the flower meristem and in the first bract primordium (Fig. 6c, d, f, h). As the flower bud developed, the expression was maintained in all three bracts (Fig. 6h).

In the tendril primordium, which developed concomitantly with the flower meristem, *PeAP1* transcripts were also detected from the very early stages of development, and the expression was maintained throughout the whole organ in growing tendrils (Fig. 6b, c, d, f, g). *PeAP1* transcripts were also detected in young leaves, more specifically in the adaxial side of these structures, but the signal was fainter as the leaves grew (Fig. 6a–f). Contrary to this, in the stipules we found no sign of *PeAP1* expression (Fig. 6a–c).

**Fig. 7:**
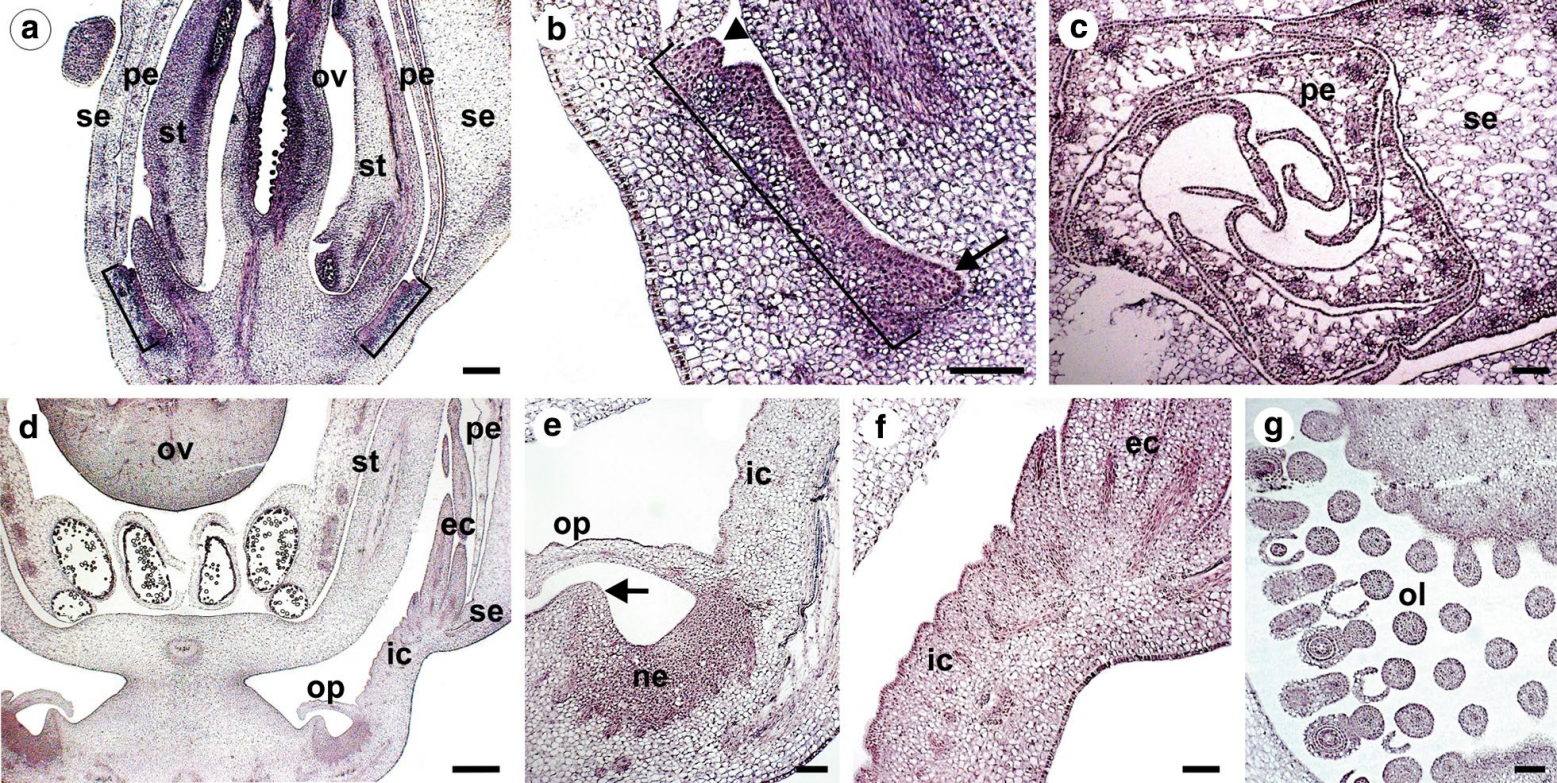
*PeAP1* expression pattern by in situ hybridization in *P. edulis* flower buds. **a**, **b** and **c** refer to flower buds of c.a. 0.5 cm and **d**, **e**, **f**, and **g** refer to buds of c.a. 1.5 cm in length. **a** Longitudinal section showing *PeAP1* expression in ovary (*ov*), stamen (*st*) and in the region where the corona filaments, as well as the operculum, will develop (*brackets*). A magnification of this region is shown in **b**, where it is delimited by the *bracket*. The *arrowhead* indicates a group of cells where a corona external filament is starting to develop, and the *arrow* indicates the region from where the limen will emerge. **c** Transverse section of a flower bud showing expression of *PeAP1* in sepals (*se*) and petals (*pe*). **d** An older flower bud, where the expression of *PeAP1* is no longer present in stamen and is weak in the ovary. At this stage, the internal and external corona filaments (*ic* and *ec*, respectively), as well as the operculum (*op*), have initiated their growth. In **e** and **f** the cells that will form the flower nectary (*ne*), as well as the internal and external corona filaments (*ic* and *ec*, respectively), the operculum (*op*), the limen that is starting to protrude (*arrow* in **e**) maintains *PeAP1* expression. **g** ovules (*l*) of flower buds of 1.5 cm still present hybridization signal. *Bars*: **a** = 50 µm; **c**, **d** = 200 µm; **b**, **e**, **g**, **h** = 100 µm

Additionally, *PeAP1* expression was also examined during the development of *P. edulis* flower buds by in situ hybridization, in order to determine the site and timing of expression of this gene in the floral organs, including the corona and the membranes that constitute the flower nectary system (operculum and limen). In flower buds (excluding the bracts) of approximately 0.5 cm in length, the petals, sepals, carpels and stamen primordia have already emerged, but not the corona filaments or the operculum and limen (Fig. 7a). At this stage, the corona can only be distinguished by a region of dividing cells between the petals and the stamen (Fig. 7a, b). In this developing corona region, the cells distally located from the base of the flower will form the external corona filaments and the ones proximally will form the operculum (Fig. 7b). The androgynophore column, which elevates the stamens and carpels, develops only at latter stages of the flower bud development, and therefore is not formed within flower buds that are 0.5 cm in length. In these flower buds, we also observed transcripts of *PeAP1* in the petal and the sepal tissues, although the ovules and the microspores also presented hybridization signal (Fig. 7a, c). Strikingly, *PeAP1* expression is observed throughout the entire group of cells that will form the inner and outer corona filaments, as well as in the developing operculum (Fig. 7a, b).

In 1.5-cm flower buds, the inner and outer corona filaments have already differentiated (Fig. 6d). At this stage, *PeAP1* expression in the corona filaments remained, but became more restricted to the epidermal cell layers of the outer and inner filaments (Fig. 7e, f). Additionally, the operculum and the limen have also already developed, and *PeAP1* transcripts were detected in these organs as well (Fig. 7e). *PeAP1* is also expressed in dense group of cells at the base of the flower, between the operculum and the limen, which corresponded to the region that will form the floral nectary (Fig. 7e). In 1.5-cm flower buds, the expression in ovules could still be detected in some cells of the ovule integuments, but it was no longer observed in microspores (Fig. 7d, g). Transcripts were also not detected in other parts of the stamen, such as the filament and the anther (Fig. 7d). The results for the sense controls are shown in the Additional file 3: Fig. S1.

### *PeFUL* has a broad expression pattern

**Fig. 8:**
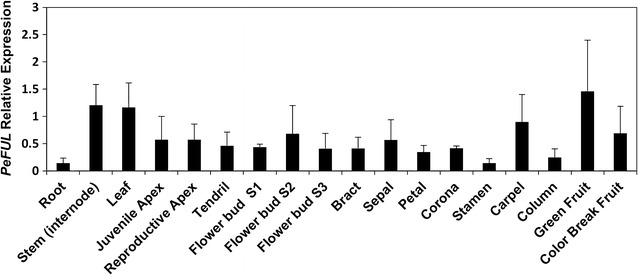
Expression profiles of *PeFUL* in different *P. edulis* vegetative and reproductive tissues by RT-qPCR. The relative expression levels were normalized with the expression of the constitutive *PeCAC* gene. The *bars* refer to the standard error of three biological replicates

The expression pattern of *PeFUL* was also investigated in *P. edulis* vegetative and reproductive tissues. The analysis of *PeFUL* expression by RT-qPCR showed that this gene has a broad expression pattern, with transcripts detected in all tissues analysed (Fig. 8). Among the vegetative tissues, stem and leaves showed the highest expression of *PeFUL* (Fig. 8). Contrary to *PeAP1*, the apices at the juvenile and reproductive stages presented similar *PeFUL* expression levels. Among the reproductive tissues, *PeFUL* expression was highest in ovary and fruits (Fig. 8).

Corroborating our RT-qPCR analysis, no differential expression was observed for *PeFUL* in in situ hybridization experiments in the organ primordia and meristems in the reproductive apices [Additional file 3: Fig. S2]. We also investigated the in situ localization of *PeFUL* in flower buds. Transcripts were also detected in the developing stamen, especially in the tapetum cells and microspores, in carpels and in the region that further originates the corona and the operculum [Additional file 3: Fig. S2].

## Discussion

*P. edulis* plants have clearly distinct juvenile and adult phases. The main differences among *P. edulis* life phases reflect the developmental changes in the axillary meristem. In the juvenile phase, the axillary meristems produce vegetative meristems that develop into new branches, reiterating the growth of the main axis. The switch to the adult phase occurs when the axillary meristems, in addition to the vegetative meristem, also produce a tendril. The flower meristems finally develop 1 to 3 nodes after the onset of tendril development, from the same initial group of meristematic cells in the axillary meristem. This developmental model in which flowers and tendrils are produced together is a common feature in Passifloraceae that display tendrils [7, 9]. In Passifloraceae, the inflorescence type can be interpreted as an axillary cyme (Fig. 1d) [9]. In cymes, the first-order axis acquires floral identity and thus terminates in a flower, while the second-order axis can continue to produce further floral meristems and form higher-order axes or it can terminate in a flower [56–58]. In *Passiflora*, the tendril can be interpreted as a modification of the primary axis of the inflorescence, while the second-order axis produces one or more flowers, depending on the degree of reduction of the inflorescence (Fig. 1D) [9]. According to this idea, one would expect to see terminal flowers instead of tendrils in *Passiflora* species that do not bear tendrils. In fact, in at least two *Passiflora* species of the subgenus *Tetrapathea* (*Passiflora tetrandra* and *Passiflora aurantioides*) the inflorescence terminates in a flower instead of a tendril, so the oldest flower occupies the place where the tendril would develop in the main axis in closely related species [9, 59]. Our results show that the tendril is the first organ produced by the axillary meristem during the switch to the adult phase, which, at least morphologically, would be another indication that the tendril corresponds to the primary axis of the *Passiflora* inflorescence.

The ultrastructural analysis shows that the tendril development is initially separated from the development of the flower. However, flower induction happens shortly after the formation of the tendrils, and once reproductive state is induced, flower buds will always accompany the tendril. Considering the spatiotemporal pattern of the tendril development, and the inflorescence architecture in other *Passiflora* species [9], it is likely that the tendril is part of the inflorescence. This indicates that either the switch to floral identity occurs as soon as the tendril develops, but further flower formation is suppressed in the tendril, or the expression of a flower meristem identity gene occurs exclusively where the flower will develop. Typical candidate genes for investigating flower meristem identity genes in *Passiflora* would be the MADS-box of the *AP1*/*FUL* clade.

The MADS-box genes *AP1* and *FUL* are transcription factors generally related to the transition to the reproductive phase, and although other functions have been reported for these genes, their role in the specification of inflorescence and flower meristem identities is conserved in many species [19, 22–30]. The control of inflorescence architecture depends on the decisions of when and where flowers will be formed, which is reflected by the activity of flower meristem identity genes [56]. To understand the genetic network controlling the inflorescence architecture in *Passiflora*, we assessed the expression pattern of *AP1*/*FUL* genes in *P. edulis*. Based on the Passifloraceae inflorescence structure and in the common ontogenetic origins of tendrils and flowers, one of our aims was to see if there was a correlation with the expression of such genes and the differentiation of structures emerging from the *Passiflora* axillary meristem.

The search for *AP1/FUL* homologues in *P. edulis* resulted in one homologue of *AP1* and one homologue of *FUL*, named *PeAP1* and *PeFUL*, respectively. The phylogenetic position of the PeAP1 and PeFUL putative proteins within the eu*AP1* and eu*FUL* subgroups, respectively, confirmed the identity of these *P. edulis* MADS-box genes.

We further examined the expression dynamics of *PeAP1* and *PeFUL* in different tissues and developmental phases of *P. edulis*. The expression of *PeAP1* was positively correlated with the transition to the reproductive phase. Furthermore, both RT-qPCR and in situ hybridization showed that not only flower meristems and flower buds express *PeAP1*, as expected, but also the tendril primordia and mature tendrils. From all tissues analysed, the tendrils were the organs that showed the highest levels of *PeAP1*. This not only provides further evidence for the hypothesis that tendrils represent modified flowers, but also suggests that one or many other factors might prevent the formation of floral organs where the tendril develops.

In *Vitis* (grapevine), where a lateral meristem produces either tendrils or inflorescence branches depending on the season, *VFUL*-*L* and *VAP1*, the putative *Vitis FUL*-like and *AP1* orthologues, are also expressed in both tendrils and flowers [2, 5]. We observed that the *PeAP1* is highly expressed in the tendril primordia, suggesting that both *Vitis* and *Passiflora* tendrils use part of the genetic programming of flower identity. Even if *Passiflora* and *Vitis* tendrils are modified inflorescences or flowers, *AP1* and *FUL* are not the genes that alone would be sufficient to induce the development of a flower, as we have shown above that such genes are expressed in tendrils from early through to late stages, but flowers were not formed. Therefore, other gene(s) might block the development of flowers in *Passiflora* tendrils or alternatively induce flower development laterally to the axillary meristems, giving rise to proper flowers. One possibility is the involvement of polarity genes in *Passiflora* flower and tendril development, such as *YABBY* genes, which in *Arabidopsis* establish abaxial identity and control inflorescence and flower architecture [60, 61]. Mutations in the *YABBY1* gene cause conversion of flowers to filamentous structures even when *AP1* is strongly expressed [61].

We also tested the expression of *PeAP1* in *Passiflora* floral organs in order to see how the sites of expression might correspond to possible functions in the identity of organs that characterize the *Passiflora* flowers. All *Passiflora* species are characterized by the presence of non-stereotypical floral organs, such as the corona, the operculum and the limen, which conceals the nectary chamber, and the androgynophore (Fig. 1a–c) [1]. These traits can be morphologically very diverse with different biological roles among *Passiflora* species, thus having a profound impact in the interaction with pollinators, and in the co-evolution process. Elucidating the genetic control of the distinct *Passiflora* floral organs is important to understand how these organs were specified during the evolution of this genus. One of our aims was also to propose a possible role for *AP1/FUL* genes in the generation of such evolutionary novelties in *Passiflora*.

Our expression analyses showed that *PeAP1* is highly expressed in the bracts, sepals, petals and in the corona, from early developmental stages until anthesis. The expression of *PeAP1* was also observed in the operculum and limen. Previously, Hemingway et al. [47] suggested that the corona of *Passiflora caerulea* is homologous to stamen, as it expresses, although weakly, both B- and C-class genes, which are traditional regulators of stamen identity. Although the corona expresses B- and C-genes, its expression pattern is different from that observed for stamens. In *P. caerulea,* the expression level of *PISTILLATA*, a B-class gene, is much stronger and stable in stamens than in the corona. In addition, our results show that *PeAP1* expression pattern is also different comparing the corona and the stamen. *PeAP1* is expressed in the corona throughout its whole development, which contrasts with its expression in stamen, where transcripts are detected mainly in initial stages of flower bud growth. The development of the corona also differs greatly in time and pattern from the stamen or any other floral organ. Ontogenetic analysis of flower buds of *Passiflora* showed no solid evidence for corona and stamen homology [8]. Additionally, the development of the corona is dependent on meristem expansion in the flower receptacle, where the filaments start to grow after all other floral organs have developed, without any clear phyllotactic pattern correlating it with the perianth or androecium [45]. The observation of intercalary meristem expansion prior to the corona formation correlates with the expression of Pe*AP1* and the conserved role of eu*AP1* in flower meristem identity. Based on our results and also on the nature of the *Passiflora* corona development, we believe that the corona is rather a novel, *sui generis* structure, as suggested by Bernhard and Claßen-Bockhoff and Meyer [45, 46].

The expression of *PeAP1* in early stages of stamen and corona development coincides with the reported expression of *P. caerulea* AGAMOUS (*PcAG*), a C-class MADS gene [47], suggesting that in *Passiflora AG* and *AP1* might not repress each other´s transcription as in *Arabidopsis* [26]. In fact, the function of eu*AP1* genes in repressing *AG* is probably not conserved and other examples of eu*AP1* being expressed in carpels have been described [37, 62].

The expression of *AP1* genes in stamen and carpels were also reporter to other species such as *Physalis*, (Solanaceae), *Jatropha curcas* (Euphorbiaceae) and also in *Vitis* [2, 37, 63]. In the majority of the cases including *Passiflora*, the expression of *AP1*is weaker when compared to perianth organs, and restricted to the early stages of the development of stamen and carpels. In *Physalis*, for example, the *AP1* orthologue *MPF3* regulates calyx identity but also promotes pollen maturation in early androecium development [37]. It is possible that *PeAP1* could perform a similar function in *Passiflora*, as transcripts are also found in microspores.

The expression of *PeAP1* in the corona filaments, as well as in the operculum and limen, also suggests novel functions for this gene in *Passiflora*. The findings indicate that *PeAP1* might have a function in the initiation of floral organs and in the further development of the perianth and the corona. The expression pattern of MADS-box genes in the corona shows that this organ retains part of the genetic programme that confers the identity of other typical floral organs, but in a different combination and expression timing and levels. Taken together, our data suggest diversification of eu*AP1* function in *Passiflora*, driven by distinct selective pressures, such as the presence of different types of pollinators.

In contrast to *PeAP1*, *PeFUL* presented a broad expression pattern throughout the whole apex, and was not restricted to reproductive apices, as its expression was also detected in juvenile apices. Additionally, *PeFUL* expression was observed in all the other organs analysed, although in different levels. Differently from *PeAP1*, *PeFUL* expression was detected in both juvenile and reproductive phases. Such extensive expression pattern is indeed more common in eu*FUL* clade than in the eu*AP1* clade [62]. The expression of *FUL*-like genes is reportedly even broader, being present in roots, stem, leaves, reproductive meristems, flower organs and fruits. It is believed that after the duplication that generated the eu*FUL* and eu*AP1* clades, the eu*AP1* clade became restricted to reproductive tissues in most cases, whereas eu*FUL* genes tended to keep the *FUL*-like broad expression pattern [29].

The eu*FUL* and *FUL*-like genes have been shown to be implicated not only in the transition to flowering but also in regulating leaf development, cell differentiation during *Arabidopsis* fruit development and the ripening process of fleshy fruits [19, 40, 42, 43]. In tomato, for example, two *FUL* paralogs, *FUL1* and *FUL2*, are involved in fruit ripening by regulating carotenoid accumulation and ethylene production [42, 64]. In bilberry (*Vaccinium myrtilus*), the *FUL* ortholog *VmTDR4* regulates anthocyanin levels during the ripening of the berries [41]. The expression of *PeFUL* in *P. edulis* carpels and fruits suggests that it might play a role in *Passiflora* fruit development. Further investigations of the role of *PeFUL* in fruit development could be of interest considering that the two main varieties of *P. edulis* produce either yellow or purple passionfruits, and are widely grown as fruit crops in several countries [65].

## Conclusions

The combined analysis of spatiotemporal development of *P. edulis* organs from the axillary meristem and *AP1*/*FUL* gene expression provide new molecular evidence that the tendril in *Passiflora* is a modification of the reproductive shoot. Our results show that *Passiflora* tendrils appeared during Passifloraceae evolution using part of the flower-related genetic developmental programme. This would thus point to the convergence of similar developmental processes involving the recruitment of genes related to flower identity in the origin of tendrils in at least Malpighiales, Vitales and Cucurbitales. Furthermore, the expression of eu*AP1* genes during the development of the *P. edulis* corona indicates this gene class might contribute to the structural diversification of flower morphology in the genus. The potential roles of *PeAP1* and *PeFUL* during *P. edulis* flower and fruit development might also contribute to passion fruit breeding in the future.

## Additional files

**Additional file 1.** PeAP1 and PeFUL sequences.

**Additional file 2.** Alignments of MADS-box proteins.

**Additional file 3.** Primers used for qRT-PCR.
