# Supplementary material for: Expression patterns of Passiflora edulis APETALA1/FRUITFULL homologues shed light onto tendril and corona identities
Source: EvoDevo. 2017 Feb 2;8:3. doi: 10.1186/s13227-017-0066-x (PMC5290658; doi:10.1186/s13227-017-0066-x)
Supplement: Supplementary file 1 — Additional file 1. PeAP1 and PeFUL sequences. [file 13227_2017_66_MOESM1_ESM.docx]

**Additional file 1**

# “Expression pattern of *Passiflora edulis* APETALA1 / FRUITFULL homologues sheds light onto tendril and corona identities”

Livia C. T. Scorza^1, 2^; Jose Hernandes-Lopes^3^, Gladys F. A. Melo-de-Pinna^3^, Marcelo C. Dornelas^1*^

^1^Universidade Estadual de Campinas, Instituto de Biologia, Departamento de Biologia Vegetal, Rua Monteiro Lobato, 255, 13083-862 Campinas, SP, Brazil.

^2^Current address: Institute of Molecular Plant Sciences, University of Edinburgh, Max Born Crescent, King’s Buildings, Edinburgh EH9 3BF, UK

^3^Universidade de São Paulo, Instituto de Biociências, Departamento de Botânica, Rua do Matão 277, 05508-090 São Paulo, SP, Brazil

***Corresponding author**: Marcelo Carnier Dornelas

**email:** [**dornelas@unicamp.br**](mailto:dornelas@unicamp.br)

**Nucleotide sequences of *PeAP1* and *PeFUL* including 5’ and 3’ untranslated regions (UTRs)**

**5’ UTR – START CODON – CDS – STOP CODON – 3’UTR**

**>PeAP1 ATCTCTCTCTCTCTCTCTCTCTCTCTCTCTCTCTCTATATCTCTCTGCGTGTGTTCTGTTTTCCTTTTTTATATACCGCTCTCTTATGGTTTGGTGTTTTTTTGGTTTCTCTTGGGTTTTGTTTGTTGGGTTATTTTGAGGAAACAAAGATTTTTGGAGTGGGAAAGGAAACAGATAAAAAAAAAAAAAAAGAAGAAATGGGAAGGGGTAGGGTTCAGTTGAAGAGGATAGAGAACAAGATCAACAGGCAGGTGACATTTTCCAAAAGGAGAGCTGGGTTGTTGAAGAAAGCTCATGAGATCTCAGTCTTGTGTGACGCTGAGGTTGCTTTGATTGTCTTCTCCCATAAGGGAAAGCTCTTTGAATACTCCACTGATTCGGGCATGGAGAAGATACTCGAACGCTACGAGAGGTATTCATATGCAGAGAGGCAGCTGGTTGCCACGGACACCGATTCAGAGGGCAACTGGGCCTTGGAGTACAAAAGGCTGAAGGCAAAAGTTGAGCTTTTAGAGATAAACCACAGGCACTATCTGGGAGAAGATCTCGAGTCTGTGAGTCTCAAGGAGCTACAATCCTTGGAACAACAACTTGATGCTTCCCTGAAGCATATTCGGTCAAGGAAGAACCAACTGATGTACGAGTCGATCTCTGAACTTCAAAGGAAGGAAAAGGCAATACAGGAGCACAATAACCTATTAGAAAAGCAGATCAAGGAGAAAGAGAAGGTGGTGGTCCAACACTCACTTTGGAATCAGCAAAGTCATGACCCTAGTGCCTCCTCGTTTCTTTTGCCACAGCCAGTCCTCCCGTGTTTAAACATTGGTAGCACTTATCAAGAGGATGCTTCAGATCAAGCAAGGAGGAACGAGCTTGACCTTACATTGGAACCAATATATTCATGTCATCTAGGATGCTTTGCTGCATGATTGTTTGAAGAAACAACAAAGCATGTAGCCGCAAGCTTTTTATGGACTCTATTTTTTTTTGGCATAAACGCCCATGCGATGAGAAGTATTTTTACTTGTATTTTCATGTACTACTCATGCACTCATCATGCTTTTCGGGCAAAAACACTCATCTATCTTTATGGTATGTAAATTGTTGAAACCTTGATTATATGTTTTAATTTGTGGTCTTTGGTTATTTAAA**

**>PeFUL**

**GTATGCTCTTTTTTTTTTTTTATTTTTTATTTTGGTTCTTCGTTTGTTGTCTTCCTCTGCTGACTCTTTTCTCTAAGTGGTGGGCTTGTATATGTTGTACATAAAAGCTTGAACTTTGTGGGGGATATTTTTGTTTCTTGTAGGAGGAGGACGAGAGGGAAAGAGAGTCGTGGTTAATAACATGGGGAGAGGTAGGGTTCAGTTGAAGAGGATTGAGAACAAGATCAATAGACAGGTGACGTTTTCCAAGAGAAGGACAGGATTGTTGAAGAAGGCGCACGAGATCTCTGTGCTCTGTGATGCTGAGGTTGGTTTGATCATCTTCTCCGCCAAAGGGAAGCTTTACGAGTACTCTTCAGATTCATGCATGGAAAAGATCCTTGAACGTTATGAGAGATACTCATATGCTGATAGGCAGATGCTTACAAGTGATGCCGATACAAATGGTAGTTGGAGTTTGGAGCATGCAAAGCTCAAGGCTAGGATGGAGATTTTACAAAAAAACCAAAGGCATTTTATGGGAGAAGATCTCGAGTCCTTGAGCCTCAAAGAGCTTCAAAATCTGGAACAACAACTTGATTCTGCAATAAAACATGTCAGATCAAGAAAGAACCAATTGATGTTCGAATCGATTTCAGAGCTGCAAAAGAAGGATAAGTCGTTGCAGGAGCAAAACAACAATCTTGCGAAGAAGATCAAGGAAAAGGAAAAAGAAAAAGCAATGGCTCGGCAAGAAAATCCTGCCTTGGATGCATCGACTCTTCTTCTAGAACAACGGCTTCAACCTTCAAATAGCATAAGCATCCCGGACGCAAGAGGCGGGACATGTGAGGATCCAGTGAGTCCAACGCACCATCGAGTCAATGCACTCTTGCCGGCCTGGATGGTTCGCCACCTCAATGACTAAGCAACAAGAAGGAATAAAGCTGTATGTGATTCAAATAATTTGGTCCGTGCAAACTAATTGAAACATTCAACAAAGAAATATAAACTTAGAAATATGTGGTTGGGTCTGATGAGAGTCCAATTTCGAACGATTCTTCCTTACTATATAGTCCCAAAAACTATGTATCTATCTTTATAGATTTTGGGATGATGTTCTGAAACAATGTAAACTAGTCTGCTTTTAGTAATCTACTTACGGTAATTAATGATGGTTTAAAAAAAAAAA**
