# Supplementary material for: Expression patterns of Passiflora edulis APETALA1/FRUITFULL homologues shed light onto tendril and corona identities
Source: EvoDevo. 2017 Feb 2;8:3. doi: 10.1186/s13227-017-0066-x (PMC5290658; doi:10.1186/s13227-017-0066-x)
Supplement: Supplementary file 3 — Additional file 3. Primers used for qRT-PCR. [file 13227_2017_66_MOESM3_ESM.docx]

**ADDITIONAL FILE 3**

# “Expression pattern of *Passiflora edulis* APETALA1 / FRUITFULL homologues sheds light onto tendril and corona identities”

Livia C. T. Scorza^1, 2^; Jose Hernandes-Lopes^3^, Gladys F. A. Melo-de-Pinna^3^, Marcelo C. Dornelas^1*^

^1^Universidade Estadual de Campinas, Instituto de Biologia, Departamento de Biologia Vegetal, Rua Monteiro Lobato, 255, 13083-862 Campinas, SP, Brazil.

^2^Current address: Institute of Molecular Plant Sciences, University of Edinburgh, Max Born Crescent, King’s Buildings, Edinburgh EH9 3BF, UK

^3^Universidade de São Paulo, Instituto de Biociências, Departamento de Botânica, Rua do Matão 277, 05508-090 São Paulo, SP, Brazil

***Corresponding author**: Marcelo Carnier Dornelas

**email: [dornelas@unicamp.br](mailto:dornelas@unicamp.br)**

**Table 1. Primers sequences used in this study for RT-qPCR and RNA probe synthesis**

| Gene | Primer sequence | | Fragment  size | Use | Primer  Efficiency (%) |
| --- | --- | --- | --- | --- | --- |
| *PeAP1* | FW | CACTGATTCGGGCATGGAGA | 559 | RNA probe | _ |
|  | RV | TCATGCAGCAAAGCATCCTA |  |  |  |
|  | FW | ACCCTAGTGCCTCCTCGTTT | 161 | RT-qPCR | 86 |
|  | RV | TCATGCAGCAAAGCATCCTA |  |  |  |
| *PeFUL* | FW | GTGCTCTGTGATGCTGAGGT | 615 | RNA probe | _ |
|  | RV | GTCATTGAGGTGGCGAACCA |  |  |  |
|  | FW | ATAAGCATCCCGGACGCAAG | 74 | RT-qPCR | 86 |
|  | RV | AGTGCATTGACTCGATGGTG |  |  |  |
| *PeCAC* | FW | TCAAGAGGGAGTGCGTTCAC | 90 | RT-qPCR | 99 |
|  | RV | CAACCAACAGCGCCTGTAAC |  |  |  |

**Supplementary Figure 1**

**
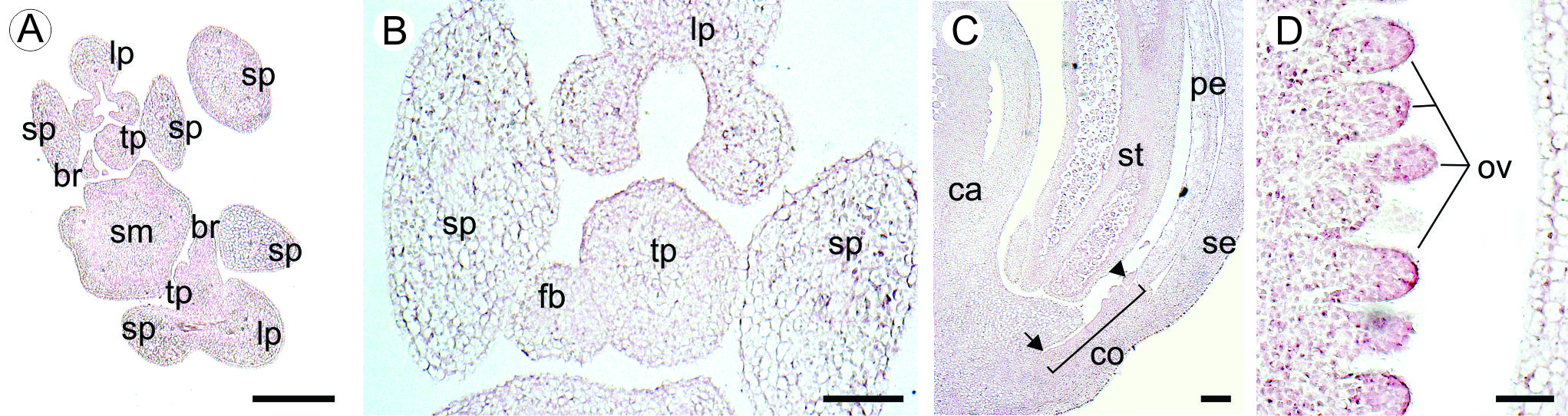
**

**Fig S1** ***In situ* hybrization of *P. edulis* apices and flower buds using the *PeAP1* sense control probe**. **A** Cross section of a reproductive apex showing the shoot meristem and leaf, stipule, tendril and bract primordia (sm, lp, sp, tp, bp, respectively). **B** Detail of a tendril primordium and a flower bud (fb). **C** Longitudinal section of a flower bud of 5 mm in length showing flower organs such as sepals (se), petals (pe), stamen (st), carpels (ca) and the region where the corona will develop (co; bracket). The arrowhead indicates the region where the external corona filaments will develop and the arrow indicates the region where the operculum will develop. **D** Ovules (ov). Bars: A= 100 µm; B-D = 50 µm.

**Supplementary Figure 2**

**
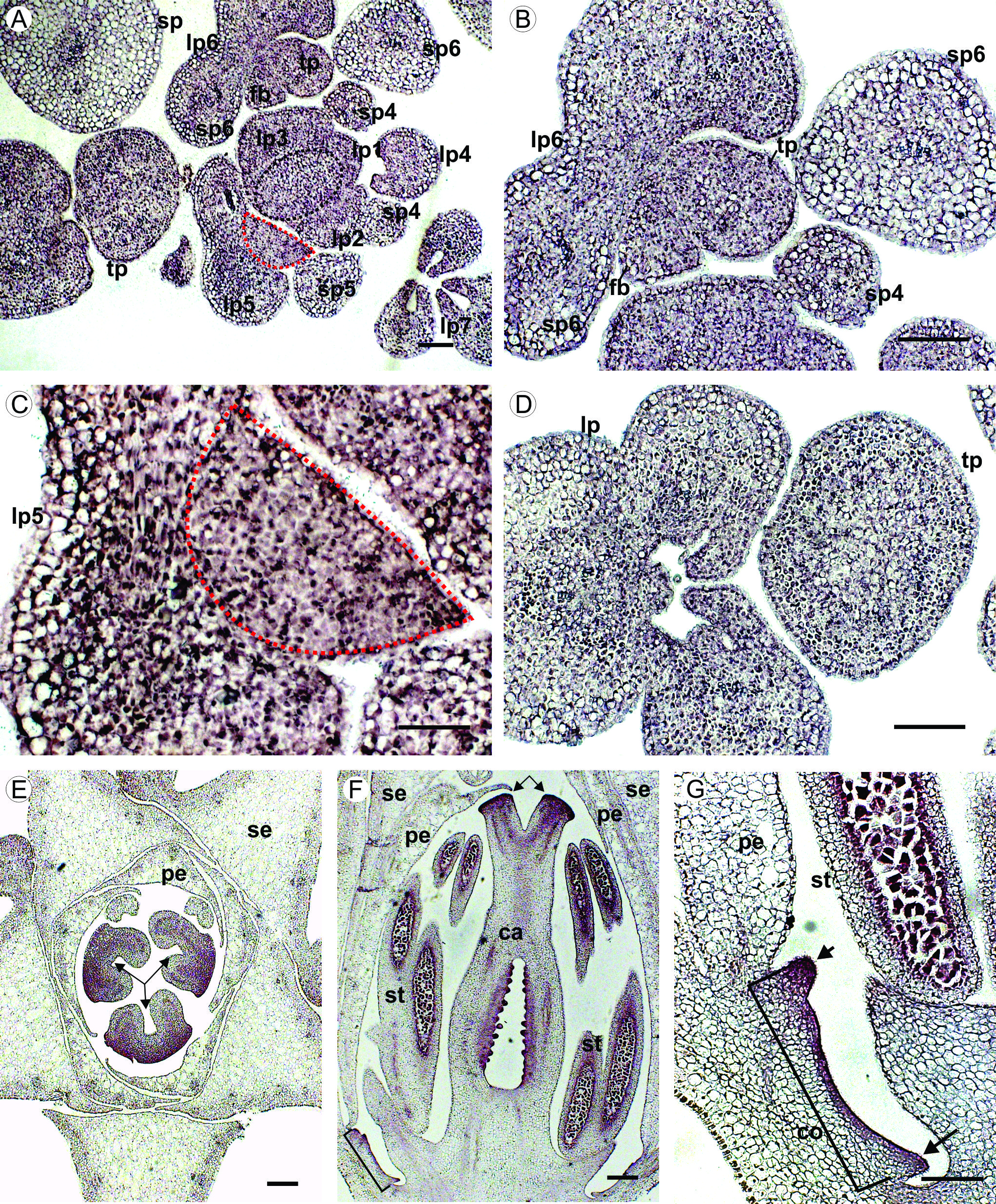
**

**Fig S2**  ***PeFUL* expression pattern by *in situ* hybridization of shoot apices of plants in the reproductive stage** **(A-D) and in flower buds (E-G).** **A-D** Cross sections of the apex showing the leaf primordia (lp) developing in a spiral phyllotaxy from the apical meristem (black dotted circle in **A**). The flower bud and the tendril primordium (fb and tp, respectively) can be seen in the axil of the sixth leaf primordia (lp6) in **A** and **B**. The axillary meristem is delineated by the red dotted semi-circle in **A** and highlighted in **C** (xm). **D** A leaf primordium and a tendril primordium showing *PeFUL* expression. **E-G** Flower buds of c.a. 5 mm in length. **E** Cross section of a flower bud showing petals (pe), sepals (se) and the three stigmata (arrows). *PeFUL* expression is mainly in the stigmata. **F** Longitudinal section of the flower bud showing *PeFUL* expression in the ovules inside the carpels (ca), in the stigmata (arrows) and in the stamen (st). In the stamen the expression is more specifically in microspores and tapetum cells. **G** *PeFUL* expression in the region where the corona will develop (co; bracket), including the upper and lower ends of this region where the external filaments (arrowhead) and the operculum (arrow) are starting to develop. Bars: A-D= 100 µm; E,F= 200 µm; G= 50 µm.
